# PSYCHOSOCIAL IMPACT OF COVID-19 ON STUDENTS AT INSTITUTIONS OF HIGHER LEARNING

**DOI:** 10.46827/ejes.v8i6.3770

**Published:** 2021

**Authors:** Elizabeth O. Akin-Odanye, Ernest Kaninjing, Roland N. Ndip, Carol L. Warren, Chioma C. Asuzu, Ivette Lopez, Charles Muiruri, Helene Vilme

**Affiliations:** 1 Department of Clinical Psychology, University College Hospital, Ibadan, Nigeria; 2 School of Health and Human Performance, Georgia College & State University, 231 W Hancock St, Milledgeville, GA 31061, United States of America; 3 Department of Microbiology and Parasitology, Faculty of Science, University of Buea, Box 63, Buea Cameroon; 4 Health Equity Consultant, University of Tennessee Health Science Center, Memphis, TN, United States of America; 5 Department of Counselling and Human Development Studies, University of Ibadan, Nigeria; 6 Division of Public Health, Department of Family & Preventive Medicine, University of Utah School of Medicine, Salt Lake City, Utah, United States of America; 7 Department of Population Health Sciences, Duke University School of Medicine, Durham, NC, United States of America; Kilimanjaro Christian Medical University College, Moshi, Tanzania; 8 Department of Population Health Sciences, Duke University School of Medicine, Durham, NC, United States of America

**Keywords:** COVID-19 pandemic, mental health, psychological distress, isolation, psychosocial care, institutions of higher learning

## Abstract

Students at higher institutions of learning are more susceptible to psychosocial problems compared to the general public. These may further be exacerbated by the measures put in place to curb the spread of COVID-19. This mixed methods study examined the factors associated with the psychosocial impact of COVID-19 on students’ financial stability, interpersonal relationships and worries related to achieving academic milestones. Data comprised of a series of closed and open-ended questions collected via Qualtrics from students in the United States and Africa (Central and West). The quantitative data were analyzed using frequency counts, percentages and chi-square, while the qualitative data was analyzed using thematic content analysis. More than 90% of the students resided in the United States, 72.5% were females and 78.4% were undergraduates. Financial hardship was experienced by 26.4% of the students, 55.8% indicated that COVID-19 negatively affected their relationship with friends and over 40% worried over delays in achieving academic milestones. Continent of residence, employment status and financial hardship were significantly associated with the negative impact of COVID-19 on one or more of the students’ relationships and with worries about achieving academic milestones. Qualitative data support the findings that financial hardship contributed to experience of psychological distress by students. It also revealed negative (compromised relationships – broken or fractured relationships and loneliness) and positive (bonding) impact of COVID-19 on interpersonal relationships. School administrators should provide students with resources to access economic relief packages and tele-counseling services to help meet their financial and psychosocial support needs amidst COVID-19.

## 1. Introduction

Preceding the COVID-19 pandemic, psychological and social problems among college and university students were already being considered a serious challenge (Salzer, 2012; Nsereko, David, Seggane, Janet, & Denis, 2014). University life marks a transitional period for students, during which some leave home for the first time, losing the parental supervision and family social support they had been accustomed to; a transition which by itself could result in psychosocial distress (Beiter *et al.*, 2015). Furthermore, students in higher institutions of learning are exposed to internal and external pressure to perform well academically and succeed despite increased academic and course workload requiring self-directed learning with effective time management skills (Othman, Ahmad, El Morr, & Ritvo, 2019; Nwachukwu et al., 2021). Students who hold part-time or full-time jobs while completing their studies can be predisposed to experiencing psychological stress due to the exigencies of balancing the demands of school and work (Carney, McNeish & McColl, 2005; Steinberg & Dornbusch, 1991). For the students that do not have gainful employment, the financial strain can lead to psychological stress impacting their studies (Liu, Stevens, Wong, Yasui, & Chen, 2019). In addition, some students encounter challenges with living arrangement, strain in forming and maintaining interpersonal relationships, which for some may lead to isolation, which in turn can increase likelihood of psychological distress (Beiter *et al.*, 2015; Liu, *et al.*, 2019). For those that thrive on social interactions with peers, when the opportunity to continue to engage socially is limited, psychological distress can also arise (Xiao *et al.*, 2019; Peloso *et al.*, 2020; Qi *et al.*, 2020).

While some level of pressure does sometimes serve as a motivation for students to meet academic demands, pressure that exceeds the students’ ability to cope can manifest itself in psychological distress (Suleyiman & Zewdu, 2018). Poor psychosocial wellbeing among university students have been associated with poor quality of life, poor sleep quality (Oh, Kim, Na, Cho, & Chu, 2019; Opoku-Acheampong *et al.*, 2017), and decreased cognitive ability leading to poor academic performance (Frazier, Gabriel, Merians, & Lust, 2019). The emergence of COVID-19 and the attendant measures to curb its spread necessitating lockdown, social distancing and transitioning to online mode of learning may have increased the prevalence of psychological distress among students.

Majority of the studies on the psychosocial impacts of COVID-19 on college students have been limited in their generalizability, reporting results from samples in single institutions only, or based on data from a single country, or several countries in the USA, Europe, Asia and the Middle East (Aylie, Mekonen & Mekuria, 2020; Sundarasen *et al.*, 2020; Wathelet *et al.*, 2020; Batra, Sharma, Batra, Singh, & Schvaneveldt, 2021); usually, with sparse data from African countries. In the current study, we describe the psychosocial impacts of COVID-19 on undergraduate and postgraduate students’ financial stability, interpersonal relationships and worries related to achieving academic milestones in various universities and colleges in the United States and three African countries - Cameroon in Central Africa, Ghana and Nigeria in West Africa.

## 2. Methods

An explanatory sequential mixed methods design was used in this study to assess the psychosocial impact of COVID-19 on students attending institutions of higher learning. Recruitment of higher institutions was done through networking with colleagues from various colleges and universities in the United States, Cameroon, Ghana and Nigeria. Students were recruited to complete a survey containing open and close-ended questions about social determinants of health (i.e., housing stability, food insecurity, food desert, health care access), access to necessary resources for successful completion of studies, social capital impact, mental health, and health activity priority (i.e., engaging in physical activity and eating healthy) using universities intranet system, classroom announcements, and word of mouth. The qualitative and quantitative data were collected simultaneously through the use of a survey that was administered via Qualtrics in English from May to October 2020. The survey took approximately 15–20 minutes to complete. This manuscript focuses on questions related to the psychosocial impact of COVID-19 (i.e., worry about achieving milestones, changes in relationships and social networks as well as an open-ended question about experience dealing with COVID-19).

### 2.1 Ethical Approval

The study was conducted in accordance with the Declaration of Helsinki, and the protocol was approved by the Institutional Review Boards (IRB) of the colleges/universities that agreed to allow their students to complete the survey. Informed consent was obtained from students before they completed the survey.

### 2.2 Data Analysis

#### 2.2.1 Quantitative

Baseline and demographic characteristics were summarized by standard descriptive summaries (e.g., means and standard deviations for continuous variables such as age and percentages for categorical variables such as gender). Chi-square was used to compare demographic differences in the psychosocial impact of COVID-19 on respondents based on age, gender, continent of residence and level in school. All statistical analyses were performed using IBM SPSS, version 21. For all statistical tests, a 2-tailed P value of <0.05 is considered statistically significant.

#### 2.2.2 Qualitative

Using NVivov.12, information from the students’ responses to the open-ended, questions were organized to identify and develop themes. A three-step process was used as proposed by Williams and Moser (2019), two coders read students open-ended responses searching for thematic connectivity leading to thematic patterns (open coding). Once themes emerged, initial codes were generated by *“segregated, grouped, regrouped and relinked in order to consolidate meaning and explanation”* as proposed by Lincoln and Guba (1985). All coding discrepancies were discussed until an initial codebook with detailed code definitions were agreed upon. Using the Axial coding technique, the responses were refined with the goal of developing major codes using constant comparison method to generate inductive codes. To conceptualize the themes that were found, selective coding approach was used to refine the codes. This permitted the generation and alignment of sub-codes with major themes. The qualitative data was used to provide context and more explanation for the results obtained from the quantitative data.

## 3. Results

### 3.1 Quantitative Findings

The study participants had an age range of 18–74, with mean of 23.34 and SD of 7.159.

Table 1 shows that 92.8% of the respondents were less than 36years old, 72.5% were female, 92.7% were in the USA, 78.4% were undergraduates and half were receiving college education funding from their parents/family.

Table 2 shows that approximately half of the students were unemployed and more than 1 in 4 participants indicated experiencing financial hardship since March 2020. Furthermore, over 50% of the students had their relationship with friends negatively impacted and greater than 80% were worried over delays in achieving academic milestones due to COVID-19.

Table 3 shows that male students were more likely to have none of their relationships negatively impacted by COVID-19 (p<0.05), while living in the US, being employed and experiencing financial hardship were significantly associated with students indicating that COVID-19 negatively impacting more than one relationship (p<0’05).

The result on Table 4 shows that more milestones’ achievement related worries was significantly associated with being an undergraduate (p<0.01), being currently unemployed (p<0.01) and experiencing financial hardship (p<0.01). Students residing in the Africa were significantly more likely to indicate having only one academic milestone achievement related worry while those in the US were more likely to indicate no worries (p<0.01).

### 3.2 Qualitative Findings

#### 3.2.1 Influences of Financial Strain on Psychological Wellbeing

Qualitative data support the findings that loss of employment and financial hardship experienced by students contributed to psychological distress. We looked at the influence of financial strain on the psychological wellbeing of our sample. Verbatim student citations are offered below.

“It’s really awful, our lifestyle changed in just a second, people are suffering, no job, no good electricity, no money.”

(Nigeria, 22years old, female, undergraduate)

“Dealing with Covid 19 has been emotional, challenging, tough and scary, everything seems to be upside down. Even the little funds I get to use for my up keep no longer have value as prices of goods and services increased so much.”

(Nigeria, 29years old, gender–other, postgraduate)

“It’s exhausting, really. It’s been horrible for everyone’s mental health, including my own, and has interfered with many individuals’ abilities to get their basic needs met.”

(USA, White, 27years old, Female, postgraduate)

“Terrible, my life has flipped upside down and I do not know how I’m going to make the next couple of months. I worry for my family as well because many are losing their jobs and may not make ends meet.”

(USA, 19years old, Female, undergraduate)

#### 3.2.2 Influences of COVID-19 on Interpersonal Relationships

We explored the context of the influence of COVID19 on student interpersonal relationships and found both negative and positive impact due to the lockdown measures and isolation. Negative impact comprised of the following themes: compromised relationships (either broken or fractured relationships), being alone, and loneliness. The theme generated for positive impact on their relationships was bonding. Sample quotes for each theme are presented below.

##### A. Compromised Relationships: Fractured

“Measures like social distancing has caused social issues among my family and friends.”

(Cameroon, 32years old, female, postgraduate)

“Increase in Work and my partner lost his job with no unemployment payment. It’s been extremely stressful and has almost dissolved our family.”

(USA, 28 years old, Female, postgraduate)

“The economic hardships to my family have caused tension and stress regarding the ability to maintain our quality of life and subsequently our relationships with each other.”

(USA, 19years old, female, undergraduate)

“My experience dealing with COVID-19 started of good and has now been challenging. This pandemic has strained the relationship between my spouse and I. That has been the toughest part of it all. I adjust well to the working and learning from home life. He found it difficult and it became abusive. We decided to separate for some time.”

(USA, 28, female, postgraduate)

“My wife isn’t working and we aren’t sending our son to daycare. While my job has not been affected because I work outside. My wife’s mental health has been affected because she’s been home for 7 months. This has resulted in increased arguments and increased home stress.”

(USA, 43years old, male, postgraduate)

##### B. Compromised Relationships: Broken

“My partner lost his job, and the stress of that added on to quarantine contributed to our separation.”

(USA, 34 years old, Female, postgraduate)

“My relationship ended earlier this year in part due to the stress of being apart during lockdown.”

(USA, 20 years old, Female, undergraduate)

“I’m more lonely than before, and broke up with my partner because of my increased work hours and because he’s been drinking more.”

(USA, 19 years old, Female, undergraduate)

##### C. Bonding

“It has strengthen the tide (tie) with my family.”

(Nigeria, 39years old female, postgraduate)

“I’ve had more time for my family.”

(Nigeria, 45years, Female, postgraduate student)

“I have become closer to friends and family because I have more time to talk to them.”

(USA, 30years old female, postgraduate)

“Overall, I have been lucky in the fact that my family and I have not lost their jobs and are able to work from home. This allowed us to spend more time together as a family and strengthened our relationship.”

(USA, 21years old, Male, Undergraduate)

The relationships of women seem to have been more negatively impacted than men’s. This is especially more depicted in the quotes from women residing in the US. Furthermore, the role of financial considerations in promoting compromised or bonding relationships appears stronger amongst students living in the US than those living in Africa.

### 3.3 Psychosocial impact of delays in achieving academic milestones due to COVID-19

We examined verbatim quotes of students to provide the context of students’ worries about delays in achieving academic milestones.

“Covid 19 pandemic has made me bored and disturbed so much on my educational career due to the delays and disruption of lectures and teaching activities.”

(Ghana, 23years old, Male, undergraduate)

“My family and I are still looking for the silver lining in all these. I had dreamt a lot about graduation since I resumed university 5 years ago, but now, there is little hope. I feel like time is slipping away from me.”

(Nigeria, 22years old, Female, undergraduate)

“Covid brought a sudden change in many aspects of my life, had plans of finishing with studies. Look for a job and help my family out, the outbreak stopped everything for a while giving me a longer time to accomplish all set goals.”

(Cameroon, 24 years old, Female, postgraduate)

“I was unable to complete my medical internship for my Speech-Language Pathology program, which was supposed to take place in a skilled nursing facility. I am nervous it will be delayed even more and that I may not graduate on time.”

(USA, 28 years old, female, postgraduate)

“My internship at a public school has been jeopardized. I must intern before graduating and I am desperately hoping the internship still goes through. I need to graduate so that I can get a decent paying job to begin paying off my student loans.”

(USA, 24 years old, female, undergraduate)

“The greatest impact on myself is the delay of my dissertation research since we were unable to come into the labs end of March-end of May. This will cause me to not graduate for an extra semester which requires registering and paying for another semester of maintenance fees.”

(USA, 25 years old, female, postgraduate)

The worries about graduation delays among students in the US was mainly related to further delay in getting a job to start offsetting student loans while for students in the African countries it was mainly about further delaying their ability to start helping the family.

## 6. Discussion

College and university students are vulnerable to emotional distress and mental illness (Downs, Galles, Skehan, & Lipson, 2018; Othman *et al.*, 2019). According to Sundarasen et al. (2020), financial constraints are a predominant stressor student encounter amidst the COVID-19 pandemic. In our study, we found that this stressor was significantly associated with negative impact on relationships and worries concerning meeting academic milestones. Students were concerned about their ability to pay study loans and meet other financial needs. Often, they experienced personal or family experience of loss of income due to being laid off or reduced work hours. Also, we found that being an undergraduate was significantly associated with more worries about achieving academic milestones compared to being in postgraduate studies. Other studies have reported decreased distress among university and college students in higher academic levels (Aluh, Abba, & Afosi, 2020; Njim et al., 2020; Votta & Benau, 2014), while some did not find any difference (Emmanuel, 2016; Opoku-Acheampong *et al.*, 2017). A possible reason for decreased worries about achieving academic milestones among postgraduate students could be that they are able to make higher wages having had a degree. Also, they are often more mature in navigating academic life due to prior exposure. However, undergraduates that may be encountering their first autonomous life are more likely to find the unexpected disruption of academic plans novel and disturbing. Furthermore, most postgraduate students may already have a stable career path they are building on. Conversely, undergraduate students are often still formulating a career path and likely to be bothered by any delay to achieve the academic success needed to create their career path. This variation between postgraduate and undergraduate students in worries about achieving academic milestones emphasizes the need that students need unique psychosocial support to help them successfully resolve their worries.

Our qualitative data results showed that relationships with friends and family were often strained, resulting in fracture or breakup due to social distancing from friends as well as increased financial pressure. Previous studies have reported that while some families, especially those with financial constraint are more vulnerable in response to challenges, others have shown resilience and were able to survive trying times (Calhoun &Tedeschi, 2014). This was further attested to by some of our qualitative data showing some positive impact on family relationships through increased opportunities to bond. This is similar to findings of other studies conducted during the pandemic (Prime, Wade, & Browne, 2020; Naser et al., 2020).

Further, residing in the US was significantly associated with negative impact on relationships due to the COVID-19 and less worries about achieving academic milestones compared to their counterparts in Central and West Africa. A possible reason for these finding may be in the cultural differences between the US and the African countries. The US have an individualistic culture while most African countries subscribe to collectivistic culture (World Population Review, 2021; Airhihenbuwa, et al., 2020). Additionally, Triandis (1993) described the prototypical relationship in individualist culture as the market place where individuals give money in exchange for goods and/or services. The relationships are emotionally distant and despite frequent interactions amongst members of the market, each member’s distinct identity is maintained. The marketplace encourages competition, and status is usually determined by individual achievement and success and not by membership in a particular group. Collectivist cultures on the other hand, are associated with low relational mobility in which relationships that are stable, strong and enduring being built on family ties and geographical areas rather than choice (Kito, Yuki, & Thomson, 2017). Furthermore, people in collectivist cultures tend to believe they should stick together through tough times and seek to preserve the harmony within the group, even if they report less intimacy with group members (Liu, Morris, Talhelm, & Yang, 2019). Thus, it has been observed that the impact of a pandemic is often greater in individualistic societies where people care less for the greater good (Maaravi, Levy, Gur, Confino, & Segal, 2021).

### 6.1 Limitations and Strengths

This study highlights the psychosocial impact of COVID-19 on college and university students’ residing in the US and Africa (Central and West). However, the findings should be interpreted with in the context of the following limitations. First, this study was cross-sectional, capturing the impact of COVID-19 for a moment in time; longitudinal impact is unknown. Second, all the measures used in this study were self-reported. As with any self-report measure, their introduced social desirability bias. However, the fact that the survey was web-based and students did not have to provide information that revealed their identity may have mitigated this issue.

### 3.2 Implications for Practice

This study has shown that financial hardship due to job loss or reduced work hours was associated with students’ worries about achieving academic milestones and with the negative impact of COVID on family relationships. To ameliorate the psychosocial impact of COVID on university and college students, higher institutions and the government should cooperate to promptly provide resources to access economic relief packages to students and their families while also opening tele-counseling lines to help provide psychosocial and informational support to students as needed.

## Figures and Tables

**Table 1:** Demographic characteristics of respondents

Characteristics	Frequency (%)
**Age categorization**	
18–35years	2,037 (92.8%)
36–50years	97 (4.4%)
>50 years	43 (2.0%)
Not indicated	18 (0.8%)
Total	2,195 (100.0%)
**Gender**	
Male	562 (25.6%)
Female	1592 (72.5%)
Non-binary	29 (1.3%)
Others	11 (0.5%)
Not indicated	1 (0.1%)
Total	2,195 (100.0%)
**Countries represented**	
USA (the 4 regions of the US were represented)	2,035 (92.7%)
Nigeria (West Africa)	61 (2.8%)
Ghana (West Africa)	33 (1.5%)
Cameroon (Central Africa)	66 (3.0%)
Total	2,195 (100.0%)
**Level/year on program**	
Undergraduate	1721 (78.4%)
Graduate levels	474 (21.6%)
Total	2,195 (100.0%)
**College education funding**	
Parent/family	1,115 (50.8%)
Scholarships	966 (44.0%)
Loans	845 (38.5%)
Employer	89 (4.1%)
Personal funds	356 (16.2%)
Other	104 (4.7%)
Not indicated	4 (0.2%)
Total	3,479 (158.5%)*

* Total exceed 100% due to respondents choosing multiple funding sources.

**Table 2:** Psychosocial impact of COVID-19 on college students

	Frequency (%)
**Employment status and financial hardship**	
Currently employed	1087 (49.5%)
Work hours reduced due to COVID-19	464 (21.1%)
Currently unemployed	1094 (49.8%)
Laid off due to COVID-19	304 (13.8%)
Experienced financial hardship since March 2020	579 (26.4%)
**Type of relationship negatively affected by COVID-19**	
Relationship with parents	762 (34.7%)
Relationship with friend	1,224 (55.8%)
Relationship with significant other	460 (2.96%)
Others	499 (22.7%)
Not indicated	367 (16.7%)
Total	3,312 (150.88%)*
**Worries related to achieving milestones during COVID-19**	
Delay graduation	962 (43.8%)
Delay completion of prerequisite/core courses	1,007 (45.9%)
Add another semester to student loans	638 (29.1%)
Others	386 (17.6%)
No worry	371 (16.9%)
Total	3,364 (153.3%)*

* Total exceed 100% due to respondents choosing multiple options.

**Table 3:** Association between college students’ sociodemographic characteristics and negative impact of COVID-19 on relationships

Sociodemographic characteristics	Number of negatively impacted relationships			X^2^	P-value
	None	Only 1	Greater than 1		
**Age**					
18–35years	564 (27.7%)	723 (35.5%)	750 (36.8%)	0.447	0.978
36–50years	24 (24.7%)	35 (36.1%)	38 (39.2%)		
>50years	12 (27.9%)	15 (34.9%)	16 (37.2%)		
**Gender**					
Male	175(31.3%)	207 (37.0%)	178 (31.8%)	9.634	**0.047** *
Female	420(26.3%)	560 (35.1%)	614 (38.5%)		
Non-Binary and Others	9 (22.5%)	15 (37.5%)	16 (40.0%)		
**Level of study in school**					
Undergraduate	458(26.6%)	622 (36.1%)	641(37.3%)	3.614	0.164
Graduate	147 (31.0%)	160 (33.8%)	167 (35.2%)		
**Continent of residence**					
North America (USA)	587(28.9%)	692(34.0%)	756 (37.1%)	38.137	**0.000** **
Africa (Central and West)	18 (11.3%)	90 (56.3%)	52 (32.5%)		
**Employment status**					
Employed currently	316 (29.1%)	358 (32.9%)	413(38.0%)	6.976	**0.031** *
Unemployed currently	289 (26.1%)	424 (38.3%)	395 (35.6%)		
**Have financial hardship**					
Yes	86 (14.9%)	209(36.1%)	284(49.1%)	81.475	**0.000** **
No	477 (32.3%)	536 (36.3%)	465 (31.5%)		

** p<0.01

* p<0.05

**Table 4:** Association between college students’ sociodemographic characteristics and worry about delay in achieving milestones due to COVID-19

Sociodemographic characteristics	Number of milestones achievement related worries			X^2^	P-value
	No worries	Only 1 worry	Greater than 1 worries		
**Age**					
18–35years	491 (24.1%)	778 (38.2%)	768 (37.7%)	8.753	0.068
36–50years	36 (37.1%)	32 (33.0%)	29 (29.9%)		
>50years	12 (27.9%)	16 (37.2%)	15 (34.9%)		
**Gender**					
Male	151 (27.0%)	204 (36.4%)	205(36.6%)	6.241	0.182
Female	383 (24.0%)	620 (38.9%)	591 (37.1%)		
Non-Binary and Others	8 (20.0%)	11 (27.5%)	21 (52.5%)		
**Level of study in school**					
Undergraduate	392 (22.8%)	642 (37.3%)	687 (39.9%)	29.104	**0.000** **
Graduate	151 (31.9%)	193 (40.7%)	130 (27.4%)		
**Continent of residence**					
North America (USA)	538 (26.4%)	738 (36.3%)	759 (37.3%)	55.770	**0.000** **
Africa (Central and West)	5 (3.1%)	97 (60.6%)	58 (36.3%)		
**Employment status**					
Employed currently	304 (28.0%)	385 (35.4%)	398 (36.6%)	13.181	**0.001** **
Unemployed currently	239 (21.6%)	450 (40.6%)	419 (37.8%)		
**Have financial hardship**					
Yes	414 (28.0%)	213 (36.8%)	274 (47.3%)	49.197	**0.000** **
No	92 (15.9%)	579 (39.2%)	485 (32.8%)		

** p<0.01,

* p<0.05
